# Dealing with insecurity: a thematic analysis of self-reflection of prospective psychotherapists

**DOI:** 10.1186/s40359-025-03694-w

**Published:** 2025-12-01

**Authors:** Esther Knichel, Carolyn Nelles, Laura Galbusera, Johannes Lindenmeyer

**Affiliations:** 1 Department of Clinical Psychology and Psychotherapy, Brandenburg Medical School Theodor Fontane, Neuruppin, Germany; 2 MAPP-Institut, Magdeburg, Germany; 3 Salus Klinik Lindow, Lindow, Germany; 4 Department of Psychiatry and Psychotherapy, Immanuel Klinik Rüdersdorf, Rüdersdorf, Germany

**Keywords:** Qualitative research methods, Thematic analysis, Self-reflection, Psychotherapy training, Insecurity, Coping strategies

## Abstract

**Background:**

Self-reflection, the ability to emotionally and cognitively reflect one’s experiences with the intention to gain new insights, constitutes a core competency of psychotherapists. However, research on content and quality of self-reflection is scarce and standardized instruments for respective assessments are still missing. The current study qualitatively explores *semantics* (core contents), *modalities* (qualitative differences) and *foci* (direction of reflection-focus) of self-reflection with the aim to operationalize and better understand its key characteristics.

**Methods:**

Forty self-reflection essays, written by 10 psychology students, were analyzed by employing a combined inductive and deductive thematic analysis.

**Results:**

In our findings, we differentiate typical *modalities*, *foci* and *semantics* of self-reflection while underlining the connections between these aspects. At the semantic level, the feeling of insecurity emerged as the most prominent impetus for self-reflection. Previous experience, patient-interaction, and external factors like time pressure, were observed to potentially influence insecurity. While assertiveness seemed to be an ineffective strategy in dealing with this feeling, tolerating it could be identified as being helpful in reducing insecurity. Additionally, we find five different modalities of self-reflection as well as inward- and outward-related reflection foci.

**Conclusions:**

Our findings offer a first operationalization of the self-reflection competency of (prospective) psychotherapists and constitute a basis for constructing an assessment rating scale. Particularly, reflecting on dealing with insecurity has emerged as a promising key for therapeutic-skill development.

## Background

In psychotherapy training the ability of being self-reflective is considered to be a key therapeutic competency (e. g., [5, 37]). Self-reflection is defined as a cyclic process in which psychotherapists first observe and critically reflect their emotions, cognitions, and behaviors to gain self-insight. Consequently, therapists might adapt their own beliefs and approaches regarding situations within therapeutic work based on these newly gained insights [18, 33]. Empirical evidence has shown that self-reflection has an important influence on therapy process and outcome. Rønnestad & Skovholt [36]⁠ conducted an interview-study with 100 counselors/therapists at different experience levels. They show that the ability and willingness to reflect on complex phenomena and processes in therapy play a crucial role in the development of the therapist’s professional self. Continuous self-reflection is seen as a requirement for an optimal learning process for all stages of professional development, whereas a lack of it might lead to stagnant or even deteriorating development [35, 36]⁠. Several reviews summarize empirical findings on the effects of self-reflection for the training and practice of psychotherapists (see e.g., [19, 21, 31]). The following positive effects of self-reflection were found: gaining of insight, self-awareness, empathy, improvement of technical and interpersonal skills, and the ability to assess one’s own competencies [19, 21]. The training of self-reflection seems to be especially important for beginner therapists, as it also stimulates the development of critical thinking as well as of ethical decision-making [31]. Importantly, therapists who are aware of their own part in therapeutic interaction can also evaluate and, if necessary, adapt their behavior or attitude [19]. Thus, the ability to self-reflect is fundamental for the therapeutic alliance, for professional growth as well as for the development of further therapeutic competencies [31]. Self-reflection does not only yield professional benefits but also has positive effects on the therapist as a person. Knapp et al. [19]⁠ point out, that self-reflection is associated with greater self-care and a lesser tendency to overestimate one’s own abilities and thus also protects against burnout symptoms. Nikendei et al. [28] could also show in their interview-study that psychotherapists have an explicit desire to reflect on the motives behind their own behaviors. Drawing on the aforementioned research, the American Psychological Association defines reflective practice as a core competency for therapists [2, 22]⁠. Accordingly, self-reflection is specified as one of the basic competencies that should be taught during the training of psychotherapists [34]⁠.

### Theories on self-reflection

The ability to mentalize is described as a superordinate construct of (self-)reflection and is conceptualized as the imaginative ability to *'conceive mental states in oneself and in other*s' [9] and to use the gained insight for the adjustment of one’s behavior [43]. Therefore, the concept of mentalization provides an important framework for the competence of self-reflection. At the same time, the latter focuses on the self, while mentalization capacity involves the reflection of the self and others. Although, evidence shows that self-reflection competence has a positive effect on therapy process and outcome, there is still no consensus as to what constitutes effective self-reflection for psychotherapists. Research on the characteristics and quality of self-reflection in the field of psychotherapy is still scarce. Even though the above mentioned studies in the psychotherapy research field support the importance of self-reflection, they did not specifically aim at the operationalization or measurement of this construct. For instance, Bennett-Levy et al. [5] conducted a qualitative study of written self-reflections of 19 trainees undertaking a Cognitive Therapy (CT) training course, which included an explicit self-practice and self-reflection component. Their research focused on what trainees experienced while applying CT-techniques on themselves rather than on self-reflection per se. The methods and/or approaches used by the mentioned studies included qualitative interview studies (this applies to [28, 36, 37]), validation of a self-rating-tool (this applies to [33]), theory-driven frameworks (this applies to [18, 34]), as well as narrative literature reviews or overview articles (this applies to [19, 21, 31]). Taken together, on the one hand these studies help to conceptualize self-reflection through theoretically driven approaches. On the other hand, they give empirical proof that (prospective) psychotherapists assess self-reflection as an important therapeutic competency and that self-reflection has an impact on other important abilities of a psychotherapist (e.g., empathy). However, qualitative research regarding the quality and content of an empirically grounded operationalization of self-reflection in the psychotherapeutic field is still missing.

In contrast, we find several theories conceptualizing self-reflection in the pedagogical field, which will be briefly summarized herein. One of the first conceptualizations was formulated by Schön [39], who distinguishes between “reflection-in-action” and “reflection-on-action”. The latter refers to reflection on past situations (with temporal distance), whereas the former means reflecting while being in a certain situation. The author highlights the benefit of the former, which allows a modification of the reflecting person’s actions when appropriate. Yet, Schön’s focus on reflecting-in-action has been criticized for not moving beyond the immediate situation [13]⁠.

Subsequent theories focus on the differentiation between self-reflective levels (we will refer to such levels also as *modalities*). Based on an empirical investigation of written self-reflections of student teachers, Hatton & Smith [15] describe four distinct levels of reflection. The first level is described as reporting a situation (descriptive writing). According to the authors, this is a basis for it, but it is not self-reflection in itself. The second level consists in explaining/giving reasons, often based on personal judgment or knowledge (descriptive reflection). The two higher levels pertain on the one hand to the assessment of the situation, e.g., by using alternative perspectives (dialogical reflection) and on the other hand to the inclusion of the broader context of the situation (critical reflection). The authors agree with Schön [39] that the supreme form of reflection is the ability to use all levels of reflection (descriptive, dialogical and critical) while the situation on which one is reflecting upon, is still taking place. Still, they also recognize the importance of reflection-on-action as a useful starting point for beginner practitioners, who cannot yet reflect “in-action”. Krieg & Kreis [20] examined the reflections in teachers’ debriefing after class. They identify six stages of self-reflection: 1. description of the situation (not considered as a reflective process), 2. descriptive reflection (includes an evaluation of the situation or the recognition of a problem), 3. explicative reflection (specifies reasons for behaviors etc.), 4. introspective reflection (pondering of different assumptions and/or relating to one’s own experiences), 5. integrative reflection (reference to scientific theories), 6. transformative reflection (gaining new ideas for future actions) – this last stage of self-reflection can occur during the whole reflection process and ideally leads to new courses of action. The authors find a correlation between higher learning success and the occurrence of higher levels of reflection such as introspective, integrative, and transformative reflection [20]. Another categorization of levels of self-reflection was developed by Ryan [38] based on the analysis of self-reflection texts, written by students at different university faculties. The author describes the following four levels: 1. reporting a situation and responding, e.g., by expressing opinions. This level is crucial for setting a clear focus and it sets a baseline of accuracy for further levels of reflection 2. relating the situation to one’s own experiences and knowledge, 3. reasoning including comprehension of the importance of the situation, 4. reconstructing and reframing knowledge by applying new ideas stemming from the self-reflection process. The levels are conceived as building upon each other. The highest quality of self-reflection is reached at the last level (“reconstructing”). Another model worth mentioning is Jahncke`s [16] four levels of self-reflection. The model is based on a review of the literature on dimensions and quality levels of self-reflections and results in a coding scale. Jahncke’s four levels are: 1. descriptive presentation (describing the situation), 2. descriptive self-reflection (assessing the situation), 3. reasoning self-reflection (giving reasons for the assessment of the situation), 4. combining self-reflection (creating links between the current situation and scientific theories, previous experience and knowledge). In contrast to the other models the author distinguishes further dimensions: the *focus* of reflection can be on the reflecting person’s ‘outside’ (external factors, e.g., circumstances) or ‘inside’ (internal factors, e.g., behavior) as well as on the past or the future. The transfer of experience from the past to the future is emphasized as being crucial for learning processes. A good self-reflection includes all the above-mentioned levels of reflection as well as all dimensions.

The outlined pedagogical theories distinguish hierarchical levels of self-reflection that differ in their quality and are built up on each other. A high-quality self-reflection is generally identified in higher levels of self-reflection (such as “combining”, “reconstructing” or “integrative reflection”) and most of all in the combined use of the different levels. In contrast, *only* describing a situation usually does not count as part of the self-reflective process but can serve as a mere building block. All mentioned authors emphasize the need to support and train the ability to self-reflect as higher levels of self-reflections are not part of our natural thinking patterns⁠.

### Aims of the study

All conceptualizations of self-reflection, as well as the coding and rating scales developed for its assessment (see e.g., [16]), specifically pertain to the pedagogical field. Their applicability to the clinical context remains unclear.

To gain a deeper understanding of the development, qualities, and effects of self-reflection in the psychotherapeutic field, we first need an operationalization of this construct within the clinical context. This is essential for the creation of appropriate assessment instruments, which can facilitate the exploration and evaluation of self-reflective practices in psychotherapy.

In pedagogical research, self-reflection has been mainly operationalized in terms of modalities (e.g., describing, reasoning) and foci (e.g., inside, outside), i.e., the “how” of self-reflection. The *semantic* aspect of self-reflection, i.e., the patterns of meaning entailed in self-reflective narratives (the “what” or content), has been neglected so far. Yet, we allow for the possibility that the very content of self-reflection may additionally play a crucial role in the definition of its nature and quality. *Modalities* and *foci* of self-reflection are indeed tightly intertwined and might not be entirely separable from their *semantics*.

In this study, we thus investigate the *modalities* and *foci* as well as the central *semantics* characterizing self-reflections of prospective psychotherapists according to the following research questions:

1. What core *semantics* characterize the self-reflection narratives of psychology students? 2. What *foci* of self-reflection (e.g., inside, outside) can be differentiated in the self-reflection narratives of psychology students? 3. Which reflective *modalities* (e.g., describing, reasoning) can be identified in the self-reflection narratives of psychology students?

Since the aim of this study is to gain a first description, systematization and exploratory understanding of self-reflection in the context of psychotherapy training, we focused on the subjective perspectives and individual sense-making of psychology students and chose a qualitative approach.

## Methods

### Participants and data collection

As a result of the Reform of Psychotherapist Education Act, in Germany the training for becoming a psychotherapist was reorganized [11]⁠. The professional psychotherapist training is preceded by the new designed study of Clinical Psychology and Psychotherapy which concludes with the license to practice as a psychotherapist. Core competencies, such as self-reflection have a significant meaning in the newly designed Approbation Regulation for Psychotherapists [3]⁠. Respectively, the training of self-reflection is a component of university modules in the newly designed Master of Clinical Psychology and Psychotherapy at Brandenburg Medical School (BMS) throughout the whole University curriculum. We have decided to choose a cohort of students which are at the beginning of their professional career, as this offers the opportunity to use the study cohort to observe professional development over time. Correspondingly, for the present study we have selected a student cohort from the 3rd to the 5th semester of their Bachelor course of Psychology at Brandenburg Medical School. Rønnestad & Skovholt [36] categorize the professional development of psychotherapists in six phases: 1. lay helper, 2. the beginning student, 3. the advanced student, 4. the novice professional, 5. the experienced professional, and 6. the senior professional. According to Bommer et al. [6] who apply the model of Rønnestad & Skovholt to the newly designed German system, the present study cohort could be assigned to the phase 2, beginning student. This phase is characterized as a phase where the ‘*student enters professional training with aspirations to think and behave according to culturally embedded conceptions of what it means to be a therapist*’ [36]. Participants for this study were randomly selected from a cohort of 26 students (7 male, 19 female, age: M = 22,9 years, SD = 5,05). There were no exclusion criteria. During the 3rd, 4th and 5th Semesters, students take part in clinical internships (one day per week), which take place in three different psychiatric clinics. In the course of their internships, the clinical staff puts the students in charge of different tasks such as accompanying patients for walks, conducting relaxation group exercises or applying diagnostic tools. After each internship students have to hand in 3 self-reflection essays (2–4 pages) focusing on different therapeutic tasks they have been involved in during the internships. The self-reflection essays are structured by five guideline questions (see appendix). Each of the 26 students thus submitted a maximum of 9 essays over the 3 semesters. Due to some structural changes in the curricular requirements some students handed in less than 9 essays. The data pool for this study thus consisted of a total of 188 self-reflection essays, written by 26 students over a period of three semesters.

For the purpose of this study a sample of 40 texts from a subset of 10 of the 26 students (3 male, 7 female, age: M = : 22,4 years, SD = 3,01) was randomly selected. Randomly picking this subsample from the whole three-semester data pool ensures to represent possible variations in each students’ self-reflection over time. The data were collected after winter semester 2020/21, after summer semester 2021 as well as after winter semester 2021/22. On average, the self-reflection essays included 1.261 words, ranging from 606 to 3.008 words.

### Data analysis

To investigate the *semantics* central to the students ‘ narratives, when reflecting upon their clinical experiences as well as to find out about how they reflect in terms of which *foci* and *modalities* they apply, a thematic analysis [7]⁠ was conducted. Thematic analysis (TA) is a method developed for identifying themes and analyzing repeated patterns within textual data. A constructivist epistemology underpinned and shaped the use of the TA in this study [25]. The authors understand meaning and experience as a social construct, which is constructed and reproduced through a constant interaction between what is already known and ongoing influences within its agents [25].

Due to the lack of previous research on the semantic aspect of self-reflection, we explored the first research question (*semantics*) with an inductive approach. We here adopted the typical semantic focus of TA. For the second and third research questions we drew on the pedagogical literature and front-loaded the categories of *foci* and *modalities* to the analysis thus adopting an explorative-deductive approach [8]. These two categories were thus additionally considered during TA coding⁠. The TA was implemented using the qualitative analysis software MAX QDA [49]⁠.

EK conducted a TA of the whole data set, following the six analytic steps of TA ([7]: 1. getting familiar with the data, 2. generating initial codes, 3. searching for themes, 4. reviewing themes, 5. defining and naming themes & 6. producing a report). As a starting point, all texts were read to get familiar with the content. During this primary reading phase, first thoughts and ideas arising while reading the texts have been written down. Then, initial codes were generated for the whole data set. Both descriptive and interpretative codes were used [25]. In a third step, codes were clustered into themes and sub themes. The generated themes were then checked with respect to their fitting with the respective data and were revised accordingly. Finally, the themes were refined by considering the broader context of the whole data set, to ensure that they are representative for the data. This process included rereading the texts as well as comparing the results with the researchers’ initial codes. The resulting themes were sorted according to the research questions capturing the *semantics*, *foci* and *modalities* of self-reflection. To ensure trustworthiness [24]⁠, the research team applied the following steps: First, to ensure credibility the six TA steps [7] were followed closely and have been recursively implemented. Moreover, the authors utilized investigator triangulation (EK analyzed all, while a research fellow analyzed 5 essays) and consensus discussions (EK, LG, research fellow). Additionally, to ensure replicability, an audit trail reporting in detail all activities, decisions, research steps and provisional results was used to save and document all phases of the research project. The presentation of our research is based on the APA standards for reporting qualitative research (see [23]).

### Reflexivity

We are aware of several personal influencing factors regarding the data analysis. The fact that EK works as a CBT therapist shaped her perspective and expectations on what aspects of self-reflection could be important. Particularly, she became attentive to the aspects of self-awareness (thoughts, emotions, body sensations, behavior) that are typically addressed during CBT therapy. Including a research fellow (with a psychoanalytic background) and LG (trained systemic psychotherapist) in the analysis brought in more perspectives, and at the same time consolidated EK’s initial impressions. Moreover, at the beginning of the data analysis EK still worked in one of the cooperating clinics, organized students’ internships and supervised students. Her dual role of researcher und students’ tutor made it difficult for her to take an external observer’s perspective on the data. Moreover, due to her previous research experience with quantitative research methods, EK found the shift from a positivist to a constructivist perspective (which has a direct impact on the way data are coded) especially challenging. To reflect on and handle these issues EK utilized a regular exchange with the research team.

### Ethical remarks

All participants received information about the study and gave their informed consent. The data were treated anonymously and confidentially. Published data contain no quotes that could disclose the identity of any student. The study was conducted according to the national and international ethical guidelines, and it was approved by the BMS ethics committee (Brandenburg Medical School: no. E-01–20230211).

## Results

**Fig. 1:**
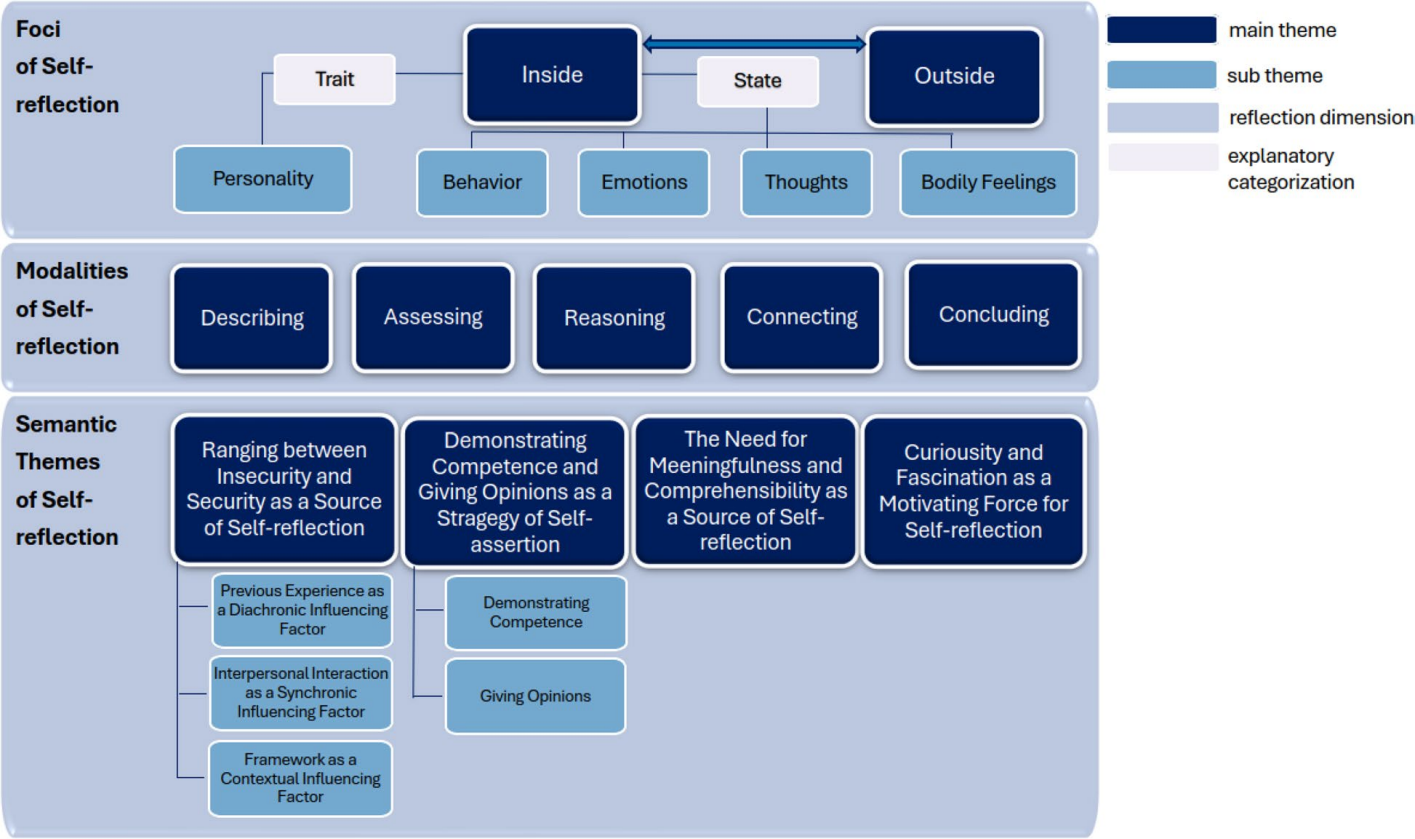
Identified themes and sub themes

The resulting themes were systematized according to the research questions into the three dimensions of *foci*, *modalities,* and *semantics*. In what follows, we describe all themes and sub themes generated in the analysis (Fig. 1).

### Semantic themes of self-reflection

We begin with the presentation of the *semantic* themes, i.e., themes pertaining to core meaningful contents in the students ‘ narratives. We carve out four central *semantic* themes that characterize students’ self-reflections: (1) Moving between insecurity and security as a source of self-reflection (2) Demonstrating competence and giving opinions as a strategy of assertiveness, (3) The need for meaningfulness and comprehensibility as a source of self-reflection, (4) Curiosity and fascination as a motivating force for self-reflection. In what follows, we present the four themes in more detail, by grounding them on textual quotes.

#### Moving between insecurity and security as a source of self-reflection

At the beginning I was nervous and insecure. In the course of the conversation, I regained the feeling of security, which I would not have expected. (Stud. 6)

The main focus of this theme is on the shift from feeling insecure to feeling secure and on the influencing factors regarding this transformative process. Indeed, we observe that the main topic of self-reflection are insecurities evoked in clinical situations. Semantically speaking, insecurity thus seems to initiate the self-reflection process.

Students described insecurity as being so challenging that their self-reflections were mostly concerned with how to handle or to get rid of this feeling. Coping strategies such as relying on theory, preparing in advance or trying to steer the situation were experienced as not being helpful in dealing with insecurity. On the contrary, enduring challenging situations and tolerating insecurity was experienced as adjuvant (for examples on hindering and helpful strategies, see quotes in sub themes below). This first theme thus emphasizes the constructive potential of insecurity (and of accepting it) as a source of self-reflective processes and thus – even if counterintuitively – as an important building block for one’s own self-confidence.

In the analysis we could also carve out factors that influenced the feeling of insecurity. These factors were clustered in three sub themes: (1a) Previous experience as a diachronic influencing factor, (1b) Interpersonal interaction as a synchronic influencing factor, (1c) Framework as a contextual influencing factor. These three influencing factors differ in the way they are temporally experienced (previous, immediate, or enduring aspects). Yet, the aspect of newness was a character shared by all three influencing factors and semantically related to the feeling of insecurity. In what follows we describe the sub themes in more detail.

##### Previous experience as a diachronic influencing factor

Across self-reflection essays we found that students’ feelings of (in)security changed according to their previous clinical experience: Lack of clinical experience yielded feelings of insecurity, whereas previous clinical experience supported a feeling of security. Interestingly, theoretical knowledge learned during lectures, in a “secure surrounding” (Stud. 5), was not experienced as having a substantial impact on students’ feeling of insecurity when dealing with challenging clinical situations. A tendency to prepare in advance or to rely on already learned knowledge when feeling insecure emerged from the narratives. However, this was not experienced as helpful in dealing with insecurity:

I tried to consider all the details we have learned: body posture, paraphrasing, relationship building. But I realized quickly that this is hard for me as a 3^rd^ semester student without much practical experience. Experience, that will occur over time (…) so that hopefully one day all these concerns will become second nature to me. (Stud. 6)

On the other hand, students mentioned previous clinical experience and practical training to be beneficial in difficult clinical situations:

The cranky comments of the patient made me feel insecure but because I have observed the reactions of therapists and doctors in similar situations, I was able to react self-confidently to his comments (…). That helped me a lot during the testing. (Stud. 3)

In this example, Stud. 3 regards themselves as being capable of reacting self-confidently due to their previous life experiences. This was confirmed by other students’ narratives: more clinical experience gave them a feeling of “calmness” (Stud. 3), “confidence” (Stud. 6) and “security” (Stud. 8). Interestingly, helpful experience was not limited to a student’s own gained clinical experience. Additionally, observing similar challenges undergone by colleagues was also perceived as being beneficial.

In the semantic analysis of text extracts, in which students reflected on their lack of clinical experience, we observe that it was essentially the aspect of unfamiliarity/newness of the situation that yielded insecure feelings. Yet, at the same time, students experienced tolerating, instead of avoiding, insecurity as helpful and as leading to more confidence in future clinical situations. Students even described feelings of pride and satisfaction after trying to face situations in which they felt insecure. Thus, not only clinical training in a narrow sense but also the enduring of previous challenging clinical experiences constitute this sub theme, which we call a *diachronic* influencing factor on (in)security. For instance, in the following quote, Stud. 6 considers rising to the challenge of tolerating feelings of insecurity as an opportunity:

This task would have been easier with a different patient. But then I would not have been able to gain the experience, to feel frustrated and to reflect on this fact, to have self-doubts regarding my competencies. I would not have been able to see the task as a chance to grow. (Stud. 6)

##### Interpersonal interaction as a synchronic influencing factor

Interpersonal interaction is presented here as a further central influencing factor on (in)security that permeates students’ narratives. Students reported that the suddenness or newness coming with the situational moment evokes challenging insecure feelings. Such insecurity is related to the experience that, in the here-and-now unfolding of an interaction, immediate reactions are required. This was thus clustered as a s*ynchronic* influencing factor. As in the sub theme above, here students also attempted to prepare in advance and to rely on previous knowledge as strategies to face insecurity. Yet, what was experienced as helpful was rather to rely on and trust in one’s own intuition as well as to engage in the interaction and adjust to it instead of trying to steer it. In the following quote a student carefully describes their emotional fluctuations in the here and now of an interaction with a patient and how they let themselves be influenced by the patient’s calmness.

Before the conversation (…) my body and my mind were relaxed (…). When the patient arrived and we entered the room together, I found I was getting nervous, and my heart raced a bit. However, this feeling changed quickly because the patient had a very calming nature, which was a bit infectious and served to calm me down quickly. (Stud. 8)

Even if it is not always the case that calmness and security are directly conveyed by patients, across these reflections we observe that engaging in the here-and-now dynamic of an interaction and – again – tolerating the insecurity related to the process, helped students to feel more grounded and self-confident.

##### Framework as a contextual influencing factor

Finally, the contextual framework that the students have to deal with appears to be another important factor affecting the feeling of (in)security. Here, with *contextual* factors we refer to external circumstances such as hospital routines or structures, time pressure, the specific role of being an intern in the psychiatric hospital, or (lacking) support by supervisors. According to the students’ reflections, these contextual and structural aspects can both be helpful and supporting, or disconcerting. Students consistently described time pressure as a hindering factor. The characteristic of this experience was that under time pressure it was not possible for students to prepare in advance and reliably predict or control a situation. Also here, what seems challenging is specifically the confrontation with the newness and suddenness of a situation:

The therapist (…) only asked two minutes before the beginning of the testing if I could conduct the testing, so I was taken by surprise. I had no time in advance to read about the test or examine it thoroughly. At the beginning of the testing, I was very insecure (...) But the further we got into the testing, the more relaxed I became. (Stud. 3)

Throughout the essays, the experience of a lack of support by colleagues and of difficult team dynamics formed another challenging contextual aspect associated with feelings of insecurity. Contrarily, the support of other clinicians appeared to help students handle insecurity. This could be the experience of being “taken by the hand” (Stud. 8) in a challenging moment or having the opportunity to exchange views with a clinician while still being in the afterglow of a demanding situation. Students described their insecurity as arising from the desire to do things right and to appear competent in their new field of work. Thus, insecurity seemed to be fed by expectations in the work environment (which were internalized by students). In the following quote, a student describes the inner conflict between wanting to meet these expectations and at the same time accepting the fact that as an intern, one does not yet have the ability to fulfill them:

The desire of an intern to appear like a professional is now paradoxical to me. At the same time, I barely know human beings who would like to willfully appear incompetent. Of course, it is important to reflect on oneself (...) but the capacity I have for the other person (the patient) would then suffer from that. (Stud. 1)

#### Demonstrating competence and giving opinions as a strategy of assertiveness

In this theme, we present a pattern of meaning that was generated across students’ self-reflections: the expression of confidence, giving opinions, and highlighting one’s own competence. In contrast to the previous theme, which included the experience of pride and confidence when facing insecurity in challenging situations (see theme 1a), the semantic core of this theme is rather assertiveness.

Here, assertiveness is differentiated from confidence as it was semantically related to a “desire… to appear like a professional”, as student 1 mentions it in the quote above, i.e., to show competence rather than feel confident and competent. Thus, in the narratives, assertiveness was related to feelings of insecurity, also yielded by the expectations from others (see also Subtheme 1c). Next to semantic codes explicitly referring to the experience of being assertive, the classification of this theme was also supported by several latent codes, which captured the implicit semantic connotation of the narrative. We further differentiate two sub themes regarding the ways in which students asserted themselves in the narratives: (2a) demonstrating competence and (2b) giving opinions. We observe throughout these narratives that when demonstrating competence and giving opinions, the students’ focus moved away from themselves (i.e., from self-reflection) and shifted to a (factual) description of good practice (as exemplified in all quotes below). Consequently, assertiveness seemed to reduce the self-reflective character of the narratives.

##### Demonstrating competence

In their self-reflection narratives, students attempted to demonstrate their competence and knowledge, e.g., by reporting about methods learned at university, which they might assume is expected from them. This is also coherent with the felt pressure to fulfill others’ expectations as shown in subtheme 1c. In the following quote a student reflects on the first time they had contact with a suicidal patient by describing the typical screening questions they asked:

I then asked him if he only dreams about suicide or if he also experiences suicidal thoughts during daytime (...). Subsequently, I asked how he would commit suicide (...) and if he already had a date in mind for when he wants to do it. Finally, I asked him if he had talked about his thoughts with others, if he had made any preparations or if he had written a goodbye letter. (Stud. 5)

This quote represents a way of demonstrating that they know how to act in the right way for this situation, more than reflecting upon their own experience in that moment.

##### Giving opinions

Within their reflections, students confidently shared their opinions and assessments on the relevant circumstances. Also here, in their given opinions we observe a shift of focus to the external reality instead of engaging in the reflection of oneself. Similar to demonstrating their competence, by giving opinions they assert themselves and appear more self-confident and secure. When giving their opinions, students engaged in considerations such as what they think raises the quality of psychiatric treatment, which interventions they find helpful, or what kind of behavior is most professional in their view. While doing so they generally took the stance of someone who knows what is right or wrong:

Regarding the concrete situation, I have the opinion that the group had a good opportunity to learn. Even though it may sound harsh, I do not think that patients should leave the hospital expecting that their environment will react with concern towards them*.* (Stud. 1)

As an extreme form of giving opinions, some students also devalued others, possibly to demonstrate their competence:

I realized in part, that I would have used different questions than the therapist and had engaged in deeper questioning (...). Moreover, I found it difficult when the therapist would interrupt the patient. In my opinion, when a patient with an eating disorder talks about eating, all information should be recorded. (Stud. 6)

#### The need for meaningfulness and comprehensibility as a source of self-reflection

Students expressed a need for meaningfulness and comprehensibility, mainly regarding the behavior of patients or clinical staff as well as in the general structures within the psychiatric hospital environment. In their accounts, students reflected upon various aspects that would cause them to question the given situations. Here they would try to find “solutions” (Stud. 1) and to “understand the patients and their concerns” (Stud. 1). The self-reflection process was thus often initiated by a need to try to understand what was yet incomprehensible:

His thoughts would not deal with suicide itself but only with the wish to not be there anymore, because he then would not have to handle the feelings of guilt. It is beyond my imagination how big the feelings of guilt must be, to have thoughts like that*.* (Stud. 5)

In this example the student acknowledges not being able to fully comprehend the patient. In the students’ narratives we observe that a lack of comprehension led to and maintained reflective processes: “The patient stayed in my mind for a very long time (…) I still don’t understand the reason for her call” (Stud. 1). Yet, the feeling of not being able to let go of an “unresolved/incomprehensible” situation could also result in rumination: “Due to the unresolved situation and the desire to find a label for the patient, I forgot (…) the task I wanted to accomplish” (Stud. 1). Thus, when the feeling of incomprehensibility becomes too much or too persistent, it might have a hindering effect on the capacity to deal with the situation, resulting in losing focus.

Yet at the same time, students reflected upon the need to accept (instead of avoid) the uncertainty related to the feeling of not-knowing:

Again, I feel puzzled. I think I am not the only one with the idea of studying psychology because I hoped that through this (applied) science I would find answers to various questions. Instead, even more questions are raised (...) instead of answering questions the essence is probably to develop an acceptance for the uncertainty. (Stud. 1)

Although looking for quick answers appeared to be an automatic impulse of many students, they also reflected upon the costs of it:”The wish to name things, to demystify them and finally get the feeling to have understood the ‘big picture’ seems to be threatening my stance of curiosity regarding the patients “ (Stud. 1).

While the feelings of incomprehensibility can challenge students and make them lose focus, giving quick answers might come with losing one’s curiosity for human beings.

#### Curiosity and fascination as a motivating force for self-reflection

Curiosity and fascination are captured as core semantic aspects of this last semantic theme. We define those, based on the narrative accounts, as being particularly relevant with regard to *motivating* the engagement in self-reflecting processes. These aspects especially occur in situations that are novel to students and often create feelings of insecurity. Thus, we observe a co-occurrence of fascination, curiosity, and insecurity.

The situation was difficult for me, because on the one hand (…) I found it fascinating that he seemed to be so convinced by his stories, as if they were reality. On the other hand, I was concerned about validating his thoughts, so that he’d lose reality out of sight even more. That is why I felt very insecure. (Stud. 5)

As deducted from the quote above, on the one hand students were fascinated by or curious about the patients they have met, their syndromes or their interactions. On the other hand, students showed fascination for observing how (“competent”, Stud. 6; “professional”, Stud. 1; “serene”, Stud. 3) clinicians handled certain situations:

While the patient had been visited during the ward round, I felt tense curiosity about how the conversation might proceed (...). I was especially curious about the interaction between clinician and patient (…). I have experienced how one (…) handles a difficult patient. (Stud. 6)

Curiosity and fascination might have the potential to act as a motivating force for self-reflection. As the quote above underlines, the way clinicians handle potentially unsettling situations seems to be particularly fascinating for students. This also relates to theme 1a, in which we could show how students’ insecurity was reduced by experiencing and witnessing the competence of more experienced therapists. Observing the clinical work of psychotherapists thus seems to both foster self-confidence and motivation.

### Foci of self-reflection

Due to their more self-explanatory nature, we will give a concise summary of themes regarding *foci* and *modalities* of self-reflection in the following. The first relevant distinction regarding self-reflection *foci* is between *inside* and *outside foci*. It is remarkable to observe that some of the students focused their reflection more on the outside world while others turned their attention back toward themselves. When reflecting on the outside, students mainly tended to focus on patients and staff, along with the circumstances in the psychiatric ward. Students also looked at the interaction between their focus on the outside and their focus on the inner life, mostly in observing how the outside world influenced what was evoked inside of them. For example, they observed how the patients’ behavior evoked certain behavioral reactions or how it affected their emotions: “In comparison to the last times I’d seen him, he appeared to be a different person. That frightened me a little bit” (Stud. 5). Focusing on the inside and the observation of how the outside influences the inside appeared to foster self-reflection. In contrast, we notice that exclusively focusing on the outside, as in the following example, seemed to prevent the self-reflective process*:*

Two other patients encouraged her that it would probably be best if she left her mother-in-law in a nursing home. This discussion helped the patient as she also said that she feels relieved not to be the only one with this problem. (Stud. 7)

Based on the narrative accounts, the inner focus can be further differentiated by focusing on *traits* or *states*. When focusing on their inner world, students would concentrate on their states rather than on their traits, meaning that they concern themselves with exploring their behavior, emotions, bodily feelings, and thoughts in the situation, rather than with their personality and how it influences their experience. The frequency of the use of those different inward related *foci* differs widely. Yet, the analysis generally shows that students focus mostly on their behavior, followed by their emotions. Few students focus on their thoughts and only a couple of them focus on their bodily feelings.

### Modalities of self-reflection

In the narrative accounts we construct five different types of *modalities* students use to reflect on situations during their internships: a. *describing*, b. *assessing*, c. *reasoning*, d. *connecting*, e. *concluding*. Students do not exclusively use one *modality* but combine them throughout their written self-reflections. Examples of the different kinds of *modalities* are shown in Table 1.

**Table 1:** Examples of modalities

***describing*: neutral description of the observed or experienced situation**
At the beginning of the testing, I introduced myself again and informed about my professional secrecy, described the procedure, and told the patient that he could ask if questions arise. (Stud. 3)
***assessing*: evaluation of situations, oneself, staff members, patients**
I would evaluate the whole conversation as professional as well as experienced. (Stud. 1)
***reasoning*: explaining reactions, feelings, etc. of oneself and others**
Moreover, the patient appeared helpless. Combined with the sympathy, it provoked a strong need to help her. The helplessness resulted from her very small and tiny figure, that she appeared so fragile and much younger. (Stud. 6)
***connecting*: drawing connections to former experiences, knowledge, etc**
In fact, I already knew about the topic, because we talked about it during a practical seminar, but it was very interesting to see how patients with psychosomatic symptoms reacted to the euthyme therapy. Therefore, it was a very informative lesson for me. (Stud. 8)
***concluding*: drawing conclusions out of the experiences made during the situation that was reflected on**
Anger and frustration are for me an indicator that I have to develop strategies to handle such and other professional challenges. My current coping strategies in this regard, like reading, are still not sufficient. (Stud. 1)

Bold Italic: type of modality, bold: description of modality, normal font: textual quotes

Interestingly, the occurrence of the *modalities* differs. The most recurrent *modality* is describing (e.g., what they do). Secondly, students use the *modalities* assessing (e.g., evaluating behaviors) and reasoning (e.g., why they would feel a certain way). Several students also occasionally use the *modality* concluding: for example, in summing up what learning experience they drew out of the described situation. Less common was the *modality* connecting (putting their experiences into context, e.g., regarding previous experiences).

Furthermore, we also observe an interesting overlap between *modalities* and *semantics*. Students would especially use *assessing* for assertiveness or for devaluing others. Both, as we discussed when looking into the *semantics*, could be viewed as possibilities to cope with insecurities.

## Discussion

The present qualitative study examines written self-reflections of a cohort of psychology students, aiming to describe and systematize relevant *foci*, *modalities* and *semantic* aspects of the self-reflection process.

**Fig. 2:**
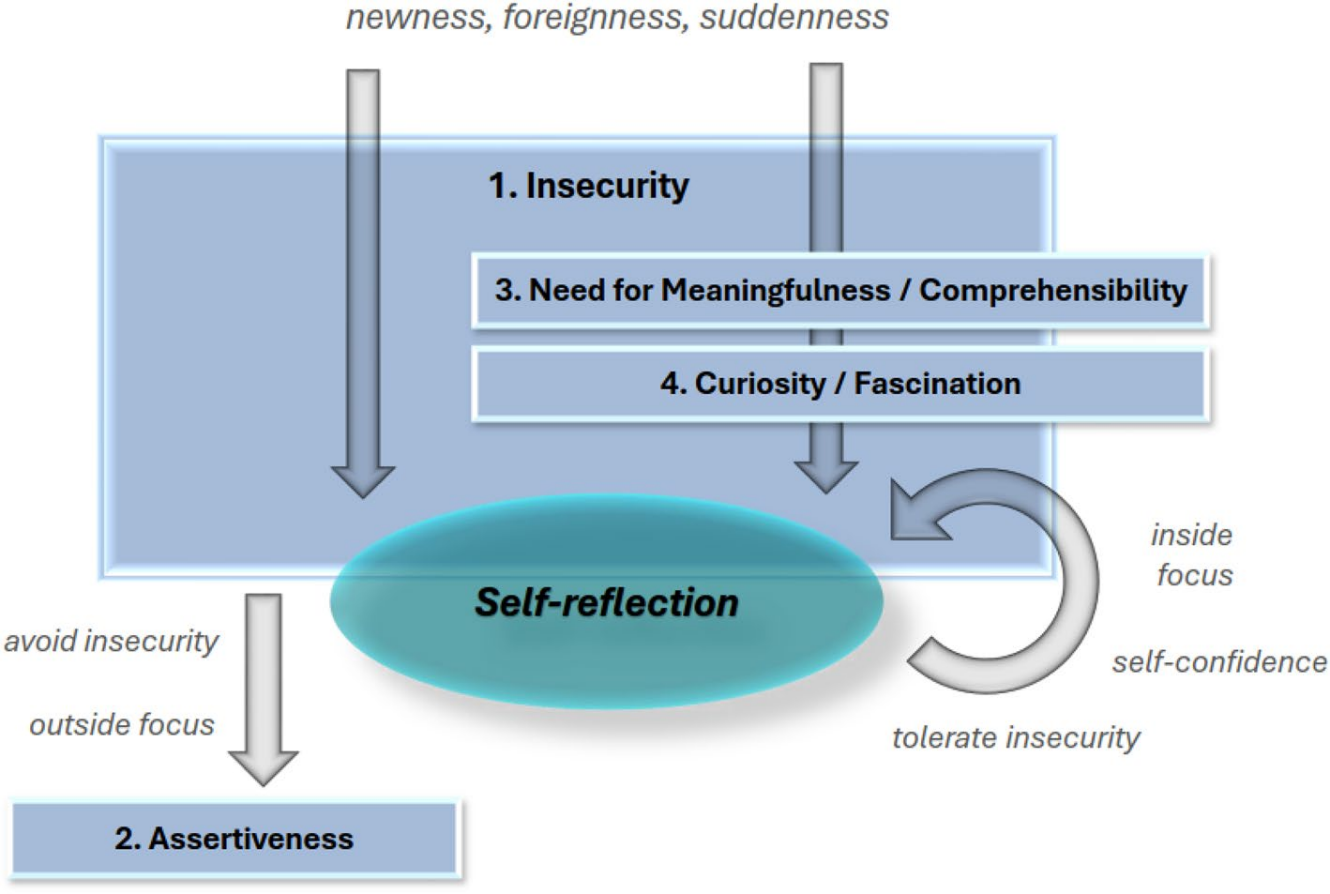
Heuristic model of the core findings. *Legend*: In the figure, we connect the core semantic themes (numbered sequentially) into a heuristic model depicting the process of self-reflection. Situational factors such as *newness*, *foreignness*, or *suddenness* give rise to feelings of insecurity (1), from which self-reflection emerges. At the same time, they fuel the search for meaningfulness and comprehensibility, as well as curiosity and fascination with the situation. Both aspects (3 and 4) serve as driving forces in the self-reflection process. Depending on how prospective therapists deal with insecurity, two different cycles can be observed: A. Avoiding insecurity leads to an outward focus, often resulting in assertive behavior (2), which in turn reduces self-reflection. B. Tolerating insecurity fosters self-confidence, enables an inward focus on internal experiences, and promotes self-reflection

Dealing with insecurity has been carved out as a core and overarching semantic theme and as the central process that fuels or thwarts self-reflection. Nearly all self-reflections in the students’ narratives express shifts on a movement between security and insecurity or can be related to this theme. The scientific relevance of our results is mainly related to two main findings: Firstly, insecurity—in its different facets—has been shown to be relevant for the initiation of self-reflection. Secondly, such an initiation of self-reflection seems to depend on the way prospective therapists cope with their insecurities. Figure 2 summarizes our findings.

Our finding that insecurity can initiate an act of self-reflection and can therefore be supportive for further professional development supports the results of previous studies [4, 27, 43, 45, 48]. Two semantic themes concerning the need for *meaningfulness and comprehensibility* as well as the *feeling of curiosity and fascination* specifically capture ways in which insecurity (e.g., the feeling of not yet knowing) can fuel self-reflection. According to Antonovsky [1], meaningfulness and comprehensibility are two necessary aspects for experiencing a sense of coherence (SOC), which is described as a resilient mindset that enables individuals to confidently navigate life’s challenges, promoting well-being and effective stress management. He describes meaningfulness and comprehensibility respectively as 1. when requirements are to be understood as challenges worth committing to 2. when events of one's inner and outer environment are predictable and explainable. When applying Antonovsky’s concept to our results, one could hypothesize that experiencing *meaningfulness* might give one a reason to better tolerate insecurity (2. challenge worth committing to). On the other hand, looking for *comprehensibility* might be an attempt to reduce insecurity by being more capable and in control (1. events are predictable and controllable). This would therefore support the finding that striving for meaningfulness and comprehensibility might help tolerate uncertainty and thus in turn foster self-reflection.

We find that feelings of *curiosity and fascination* can additionally serve as a motivating factor for engaging in self-reflection. Expectedly, we recognize that the states of *curiosity and fascination*, the need for meaningfulness and comprehensibility as well as insecurity seem usually to arise in new situations and are often experienced concurrently. As Antonovsky [1] argues, it is a typical human reaction to seek a sense of security or increased knowledge. Yet according to our results, as soon as these situations appear to be less challenging or new, they lose their potential to raise curiosity and fascination as a motivating force and in that also the potential for self-reflection seems to stagnate. This conclusion is supported by previous research, where especially new and challenging experiences were shown to prompt self-reflective processes [27, 30]⁠.

Our findings also indicate that some students experience insecure feelings as overwhelming and thus hope to eventually get rid of their insecurities. This is not surprising, as previous studies have shown that psychotherapists in training experience their first therapy sessions as challenging and distressing [36, 44, 47]. As a way of coping, we find in the analyzed accounts that students tend to compensate for their insecurities by preparing for their tasks and relying on previous knowledge. Thériault & Gazzola [47]⁠ describe this kind of impression of lacking knowledge or experience as the “empty toolbox syndrome”. Accordingly, therapists might believe they will feel more competent once they know more. Schön [40]⁠ argues that relying primarily on knowledge serves as an effective strategy for those who experience uncertainty as threatening or a sign of weakness. Yet, the author also states that it is exactly this reliance on knowledge that keeps the person away from being attentive to the moment. As our findings suggest, meticulous preparation or theoretical knowledge do not automatically reduce insecurity. It is instead prior experience that seems to have an impact on how secure students feel. The effect of experience on (in)security is supported by the research of Rønnestad & Skovholt [36], who demonstrate that insecurity occurs less as therapists become more experienced.

A second adverse coping strategy central to our results is *assertiveness*: Students present their competencies and give opinions, evaluate themselves or devalue others which leads to a shift in the focus of a reflection away from one’s own (insecure) feelings towards the outside world. We conceive of this as a way to avoid insecurity, which, interestingly enough, is also narratively related to reduced self-reflection. Thériault et al. [48] show in their interview study, that therapists apply several adverse coping strategies to compensate their feelings of insecurity, such as giving advice or sticking to theory. Similarly, as a way of mastering challenging therapeutic situations, Rønnestad & Skovholt [36]⁠ describe the concept of ‘premature closure’: By using strategies like sticking to theoretical approaches or only focusing on a certain group of patients, therapists might try to avoid being emotionally overwhelmed by reducing the complexity of the situation [36, 42]. Akin to our findings, premature closure can interrupt the self-reflection process by yielding a shift from reflection to evaluation [36, 41]. We thus hypothesize that assertiveness could be a way of premature closure and that students resort to assertiveness to deal with (or avoid) overwhelming feelings.

Nevertheless, from our findings we can conclude that insecurity is not per se deleterious. Depending on the coping strategies students adopt, insecurity could even be helpful. The current study illustrates that it is exactly the experience of accepting insecurity that seems to be the most helpful in reducing it. As previous studies could show, tolerating insecurity can also serve as a base for using constructive coping strategies (e.g., [29, 32]). In a similar manner, Galbusera et al. [10] could stress out the positive impact of tolerating insecurity for the therapeutic process. Their study implies that avoiding uncertainty (e.g., through piling up theoretical knowledge), results in a more distant relationship, whereas tolerating it leads to more closeness between therapist and patient.

Yet, our results also draw attention to potential negative effects of insecurity on self-reflection. We observe that insecurity can sometimes end up in rumination, which can lead the attention away from the interaction with the patient. In this context, previous research shows that therapists who are not able to manage insecurity might experience negative personal effects such as burn out [14]⁠ or quitting the profession [46]⁠. Finding the right balance between overwhelming insecure feelings and stagnation due to a potentially false sense of security thus appears to be very relevant for a constructive self-reflective process.

In our analysis, we can observe a complex interplay between the *semantic* aspects and other dimensions of self-reflection (*modalities* and *foci*). For example, the *semantic* of assertiveness is related to the *modality* of assessing. As such, a potential attempt to avoid insecurity is also related to a shift in the focus to the ‘outside’. Our differentiation between outside and inside *foci* matches the dimensions developed by Jahncke [16]. With the inside-outside dimension, the author differentiates between one's own behavior (inside) and exterior circumstances regarding the learning environment (outside), stressing the importance of both for self-reflection. In our analysis, we observe that if a focus on the outside steers the attention away from oneself it potentially diminishes the ability to self-reflect (i.e., possibly also affecting the *modality*). Empirical evidence indicates that focusing on the outside is more common in inexperienced therapists and fades over time [36]⁠. Interestingly, although we do not quantitatively assess the occurrence of different *foci*, a stronger (inside-)focus on emotions and behavior, and a neglect of bodily sensations, characterized the students’ narratives. Mojta et al. [26]⁠ highlight that the reflection on internal experiences including physiological reactions might be helpful for the development of self-awareness⁠. In conclusion, we assume that for psychotherapists, bodily sensations might be conceived as an important experiential aspect to be reflected upon (see [17]). Moreover, we find it remarkable that students usually do not focus on their traits (e.g., personality, behavioral patterns). Unfortunately, there is relatively little research on the question of if and how therapists’ traits influence the therapy outcome [12]⁠. More empirical evidence is needed to examine the relation between these *foci* (states-traits) and the quality of self-reflection as well as the therapy outcome.

Finally, in our findings we can differentiate five *modalities* of self-reflection: describing, assessing, reasoning, connecting, concluding. While these results represent similar self-reflection categories as discussed in the pedagogical field by Hatton & Smith [15], Jahncke [16], Krieg & Kreis [20] and Ryan [38], one important insight, emerging from our analysis, is that different *modalities* seem to relate to *semantic* aspects, which are specific to the psychotherapeutic field.

### Limitations and future directions

When interpreting the study’s results, one should consider several limitations. First, it is important to note that the analyzed self-reflection essays were part of students’ educational performance and were marked by supervisors. Moreover, students might have felt that they had to appear competent to fulfill expectations from the clinical staff. Both aspects could lead to social desirability and make students focus more on self-assertion than on self-reflection. Second, using a qualitative-explorative design, our study should be conceived as a pilot exploration, which can serve as a basis for quantitative studies on larger samples. Only such studies could potentially show how frequently the *foci*, *modalities* and *semantic* aspects derived within this work, are in fact apparent in self-reflections of prospective psychotherapists. Third, in the present work we have chosen a student cohort at the beginning stage of their career. It is important to keep in mind that our findings are not generalizable to (prospective) psychotherapists in other stages of their career. Based on our results, we cannot yet define what constitutes good self-reflection. Experimental studies are needed to support our interpretations concerning the usefulness of different self-reflection and coping styles in therapy training. Further investigations could focus on explicitly inquiring about the perspective of the reflecting individuals and their fostering professionals thereby complementing our findings on what supports and hinders the self-reflection process. Moreover, future research should investigate the question of what *modalities* contribute to the quality of self-reflection (over time), with the aim of developing a tool able to measure competency to self-reflect by means of connecting the captured *modalities* to therapeutic abilities and therapy outcome. Finally, future research could focus on the way training for psychotherapists should be designed, to support the development of self-reflection competence.

## Conclusions

To our knowledge, the present study is the first to analyze self-reflection essays in the psychotherapeutic field, particularly covering the students’ progress over the full course of their practice semesters. Our study presents important findings regarding insecurity and related feelings as sources of self-reflection as well as coping strategies in dealing with insecurity. Moreover, the findings of this study offer a basis for a first operationalization of self-reflection competency in the training of psychotherapy and constitute a basis for the construction of a rating scale for assessing it.

## Data Availability

The datasets generated and analyzed during the current study are not publicly available due to privacy and ethical restrictions but are available from the corresponding author on reasonable request.

## Appendix

### Guideline questions

During your internship you will conduct different therapeutic tasks. Please choose three of these that you consider particularly suitable for written self-reflection. For each of the three therapeutic tasks, respond to the following questions in a separate reflection:

- What was the therapeutic task (theoretical background, authors)?
- What was the purpose of the therapeutic task (which group of patients, why this group of patients?).
- How did that work (description as detailed as possible)?
- What feelings/thoughts did you have during accomplishing the therapeutic task and how did you deal with those?
- What did you learn from the therapeutic task regarding your further studies? Which conclusions do you like to draw from this for yourself?

## Abbreviations

BMS: Brandenburg Medical School
CBT: Cognitive Behavioral Therapy
CT: Cognitive Therapy
SOC: Sense of coherence
TA: Thematic Analysis
